# JUNB mediates oxaliplatin resistance via the MAPK signaling pathway in gastric cancer by chromatin accessibility and transcriptomic analysis: JUNB mediates oxaliplatin resistance in gastric cancer

**DOI:** 10.3724/abbs.2023119

**Published:** 2023-06-19

**Authors:** Suyao Li, Yichou Wei, Xun Sun, Mengling Liu, Mengxuan Zhu, Yitao Yuan, Jiayu Zhang, Yu Dong, Keshu Hu, Sining Ma, Xiuping Zhang, Bei Xu, Hesheng Jiang, Lu Gan, Tianshu Liu

**Affiliations:** 1 Department of Medical Oncology Zhongshan Hospital Fudan University Shanghai 200032 China; 2 Cancer Center Zhongshan Hospital Fudan University Shanghai 200032 China; 3 Department of Oncology Zhongshan Hospital (Xiamen) Fudan University Xiamen 361004 China; 4 Department of Obstetrics and Gynecology Zhongshan Hospital Shanghai 200032 China; 5 Department of Surgery Southwest Healthcare Southern California Medical Education Consortium Temecula Valley Hospital Temecula CA 92592 USA

**Keywords:** gastric cancer, JUNB, ATAC-seq, oxaliplatin resistance, MAPK signaling pathway, chromatin accessibility

## Abstract

Currently, platinum-containing regimens are the most commonly used regimens for advanced gastric cancer patients, and chemotherapy resistance is one of the main reasons for treatment failure. Thus, it is important to reveal the mechanism of oxaliplatin resistance and to seek effective intervention strategies to improve chemotherapy sensitivity, thereby improving the survival and prognosis of gastric cancer patients. To understand the molecular mechanisms of oxaliplatin resistance, we generate an oxaliplatin-resistant gastric cancer cell line and conduct assay for transposase-accessible chromatin sequencing (ATAC-seq) and RNA sequencing (RNA-seq) for both parental and oxaliplatin-resistant AGS cells. A total of 3232 genomic regions are identified to have higher accessibility in oxaliplatin-resistant cells, and DNA-binding motif analysis identifies JUNB as the core transcription factor in the regulatory network. JUNB is overexpressed in oxaliplatin-resistant gastric cancer cells, and its upregulation is associated with poor prognosis in gastric cancer patients, which is validated by our tissue microarray data. Moreover, chromatin immunoprecipitation sequencing (ChIP-seq) analysis reveals that JUNB binds to the transcriptional start site of key genes involved in the MAPK signaling pathway. Knockdown of *JUNB* inhibits the MAPK signaling pathway and restores sensitivity to oxaliplatin. Combined treatment with the ERK inhibitor piperlongumine or MEK inhibitor trametinib effectively overcomes oxaliplatin resistance. This study provides evidence that JUNB mediates oxaliplatin resistance in gastric cancer by activating the MAPK pathway. The combination of MAPK inhibitors with oxaliplatin overcomes resistance to oxaliplatin, providing a promising treatment opportunity for oxaliplatin-resistant gastric cancer patients.

## Introduction

Gastric cancer (GC) is the third leading cause of cancer-related death in the world [1], and the median recurrence time is 11.7 months [2]. With the development of chemotherapy drugs, the prognosis of patients with GC has considerably improved. Among these, platinum-based chemotherapy, especially XELOX (oxaliplatin and capecitabine), has been approved to prolong patient survival and suppress local recurrence in clinical practice. In particular, regimens containing oxaliplatin are likely to be more effective than those containing other platinum-based agents. However, oxaliplatin resistance has been a major impediment to sustained patient benefit from treatment and a significant cause of chemotherapy failure. There are currently no clinically applicable biomarkers for oxaliplatin response. Therefore, investigating the mechanisms of oxaliplatin resistance and identifying key biomarkers will help improve the clinical outcomes of gastric cancer patients.

In 2022, Douglas Hanahan [3] incorporated nonmutational epigenetic reprogramming into hallmarks of cancer as a supplement, thus fully recognizing its vital role in cancer initiation and progression. Epigenetics is the heritable changes in gene expression without DNA sequence changes, which may ultimately leads to changes in phenotypes. Relevant epigenetic mechanisms include DNA methylation, histone modification, noncoding RNA, and chromatin remodelling [ 3, 4] . Among them, alteration of chromatin accessibility is an essential epigenetic event. To date, growing evidence has demonstrated that alterations in chromatin structure and accessibility across the genome are significant mechanisms of drug resistance. The dynamically reorganized chromatin allows the gene regulatory machinery to open genomic regions, upregulating the gene expressions via transcription factor (TF)-mediated activation and the binding of regulatory elements. Some TFs, such as YAP [5], FOXO [6], GLI1 [7], TEAD [8] and SOX [9], have been shown to be involved in acquired resistance to multiple antitumour drugs.

Activator protein-1 (AP-1) is a ubiquitous family of dimeric transcriptional complexes, most often defined as dimers consisting of members of the Jun, Fos, Atf and Maf protein families [10]. AP-1 is involved in a wide range of physiological and pathological functions [11], and many previous studies have demonstrated that AP-1 activity dysregulation contributes to tumorigenesis, tumor progression, aggressiveness and resistance to therapy [ 12, 13] . As a key member of the AP-1 family, JUNB has been shown to promote invasion and distant metastasis in head and neck squamous cell carcinoma [14]. In addition, JUNB is involved in the regulation of bone marrow angiogenesis, thereby affecting the prognosis of patients with multiple myeloma [15]. JUNB has also been found to regulate many immune cells, such as Th17 cells [16] and regulatory T (Treg) cells [17], to mediate immune homeostasis in an interleukin-dependent manner.

In the present study, to better understand the regulatory mechanism underlying oxaliplatin resistance in GC, we investigated the systematic alterations in chromatin accessibility and gene expression through ATAC-seq and RNA-seq in both parental and oxaliplatin-resistant AGS (AGS-OR) cells. Through motif enrichment analysis, JUNB was identified as one of the key potential regulators and then validated its core role in enhancing oxaliplatin resistance in GC. Furthermore, we explored the possibility of applying MAPK inhibitors and oxaliplatin as a combination strategy to overcome oxaliplatin resistance.

## Materials and Methods

### Cell lines and reagents

HEK293T and the human GC cell line AGS were purchased from the National Collection of Authenticated Cell Cultures (Shanghai, China). 293T cells were cultured in high glucose DMEM (HyClone, Carlsbad, USA) with 10% fetal bovine serum (Gibco, Carlsbad, USA) and 1% penicillin/streptomycin (Gibco). AGS cells were cultured in RPMI 1640 (HyClone) with 10% FBS and 1% penicillin/streptomycin. All cells were cultured at 37°C with 5% CO _2_. Piperlongumine (HY-N2329) and trametinib (HY-10999) were purchased from MedChemExpress (MCE, Monmouth Junction, USA).

### Establishment of the oxaliplatin-resistant gastric cancer cell line

A previously described protocol [ 3, 18] was used to develop oxaliplatin resistance within the AGS cell line. Parental AGS cells (5×10 ^6^ cells) were initially cultured in RPMI 1640 medium with 10% FBS and 1% penicillin/streptomycin. After the cells had become adherent, they were treated with oxaliplatin at the previously calculated IC _50_ value of 5.0 μM. After 72 h of incubation, the cells were washed once with 1× PBS to remove any potential oxaliplatin released by dead cells. Oxaliplatin-free medium was added to allow cells to recover and proliferate for an additional 48 h, after which they were trypsinized and replated at 5×10 ^6^ cells/well. Forty-eight hours after plating, the cells were treated with a continuous stepwise increase in the dose of oxaliplatin in the aforementioned manner. The AGS wild-type (AGS-WT) cell line treated with oxaliplatin-free medium was synchronously passaged, and the IC _50_ values of oxaliplatin were monitored periodically throughout the development process. This process was repeated weekly for 180 days, resulting in relatively stable drug resistance. The established oxaliplatin-resistant AGS cells were named as AGS-OR cells.

### Evaluation of AGS-OR cell line sensitivity to oxaliplatin and its biological characteristics

Snow *et al*. [19] reported that the drug resistance index (RI)=IC _50_ (drug resistance cells)/IC _50_ (parental cells), and an RI>5 suggested drug resistance. In accordance with this proposed standard, the drug RI of AGS-OR to oxaliplatin was higher than 5 in this study, indicating that the AGS-OR cell line was a reliable model of oxaliplatin resistance. Compared with the parental cells, AGS-OR cells had relatively wider intercellular junctions and exhibited irregular shapes such as long strips, fusiform cells, and polygons.

### Colony formation assay

Five hundred cells were seeded per well in 6-well plates and cultured for two weeks to perform colony formation assay. The cell colonies were first rinsed in phosphate-buffered saline (PBS), and then fixed using paraformaldehyde (4%) and then stained with crystal violet (0.1%). After staining for 30 min at room temperature using a 1:1 combination of methanol and crystal violet aqueous solution (C0121; Beyotime, Shanghai, China), the culture plates were placed upside down to dry, and the cells were rinsed twice with PBS. Finally, the colonies with more than 50 cells were counted using Image J to generate a quantitative analysis.

### Transwell assay

Transwell assays were assessed using transwell plates (Corning, New York, USA). The upper chambers were separately seeded with serum-free 50,000 cells, and 600 μL of RPMI 1640 with 10% FBS was used as a chemoattractant in the lower chamber. After 24 h of incubation and fixation with paraformaldehyde (4%), the cells inside the filter membrane were stained with 0.1% crystal violet for 20 min. Five fields of view were randomly selected for each membrane under an upright microscope (Axioscope 5; Zeiss, Oberkochen, Germany), and ImageJ software (ImageJ 1.42q; National Institute of Health, Bethesda, USA) was used for subsequent analysis and comparison.

### RNA extraction and reverse transcription-quantitative polymerase chain reaction (RT-qPCR) analysis

Cells were harvested by centrifugation, and total RNA was extracted with Total RNA Kit I (Omega Bio-Tek, Winooski, USA) and RNase-Free DNase Set (Omega Bio-Tek) as described in the product manual. Reverse transcription was performed using the PrimeScript RT Master Mix kit (TaKaRa, Dalian, China). A TB Green Premix Ex Taq kit (TaKaRa) was used to conduct the qPCR assay. Primers were synthesized by Sangon (Shanghai, China) as listed in Table 1. *β-actin* was used as an internal reference, and the comparative Ct method [20] was used to quantitate the results.

**Table:** **Table 1** Sequences of primers used for RT-PCR

Name	Primer sequence (5′→3′)
*JUNB*-F	TCCAAGTGCCGAAAAAGGAAG
*JUNB*-R	CGAGTTCTGAGCTTTCAAGGT
*FGF20*-F	ATGGCTCCCTTAGCCGAAGT
*FGF20*-R	AGGAAATGCGAACCCACCTG
*PPP3CA*-F	GCGCATCTTATGAAGGAGGGA
*PPP3CA*-R	TGACTGGCGCATCAATATCCA
*MET*-F	AGCAATGGGGAGTGTAAAGAGG
*MET*-R	CCCAGTCTTGTACTCAGCAAC
*EREG*-F	GTGATTCCATCATGTATCCCAGG
*EREG*-R	GCCATTCATGTCAGAGCTACACT
*PTPRR*-F	CTGGTGTAACACATCGACCTC
*PTPRR*-R	GATTGCCATACCCGTTCCCT
*CACNA2D3*-F	TGCGGTTCGGACAAATGTCA
*CACNA2D3*-R	AATCGCCCTATCCACCAGTCA
*GAPDH*-F	CCCATCACCATCTTCCAGG
*GAPDH*-R	GAGATGATGACCCTTTTGGC

### Western blot analysis

Cells were lysed with SDS lysis buffer (Beyotime) supplemented with proteinase/phosphatase inhibitor (Beyotime), and a BCA kit (Yeason, Shanghai, China) was utilized to measure the protein concentration. Protein samples were separated by 10% SDS-PAGE and then transferred onto nitrocellulose membranes (Millipore, Billerica, USA). After being blocked in 5% skimmed milk, membranes were incubated with diluted primary antibodies overnight at 4°C, followed by washing and incubation with HRP-conjugated secondary antibodies for one hour. The protein bands were visualized with enhanced chemiluminescence reagent (Sigma, St Louis, USA). The antibodies used were as follows: JUNB (3753; CST, Beverly, USA), Erk1/2 (4695; CST), phospho-Erk1/2 (Thr202/Tyr204) (4370; CST), phospho-MEK (Ser217/221) (9154; CST), MEK (4694; CST), and β-actin (3700; CST).

### Construction of *JUNB*-silenced cells

JUNB shRNA plasmids were purchased from GeneChem (Shanghai, China). Lentivirus was produced using 293T cells and then transduced into AGS-OR cells. Stable cells with JUNB shRNA plasmids were selected using puromycin at a concentration of 1 μg/mL. Quantitative PCR and western blot analysis were used to assess *JUNB* knockdown efficiency. The shRNA targeting sequences for JUNB are as follows: shJUNB-1: 5′-CACGACTACAAACTCCTGAAA-3′ and shJUNB-2: 5′-CTCTCTCTACACGACTACAAA-3′. The sequence of negative control is as follows: 5′-CCTAAGGTTAAGTCGCCCTCG-3′.

### Preparation and data processing of RNA-seq

FastQC1 was used to evaluate the quality of FASTQ. These sequences were aligned to the human genome Hg38 with STAR [21]. With the help of Cuffdiff, the mRNA expression level of genes was quantified through fragments per kilobase million reads (FPKM) based on RefSeq gene annotation [22]. In at least one group, DEGs were selected by the criteria of q value<0.05, fold change>1.5 and average FPKM>10. To normalize and visualize the data, “ggplot2” and deepTools 2.0 were used. Using “bamCoverage” from deepTools 2.0, the format was changed from BAM to bigWig, and reads per kilobase million (RPKM) was used to normalize the data.

### ATAC-seq library preparation data processing

The ATAC-seq libraries were prepared as previously described [ 23, 24] . Bowtie2 [25] with paramete-X 2000 was used, and sequencing data were mapped with the Hg38 human genome. The removal of repeated reads depended on SAMtools [26]. Peak calling of nucleosome-free reads was carried out using the module “callpeak” of MACS2 [27]. Analysis and plotting of the distribution of paired end sequencing fragment sizes were performed using Picard Tools (v.2.2.42) and ggplot2. BAM files were transformed to bigWig format for data normalization and visualization using “bamCoverage” with RPKM. Additionally, the “plotHeatmap” and “plotProfile” scripts in deepTools [27] were used to create the average profiles and heatmaps. With the annotatePeaks.pl script in HOMER (v4.10) [28] with default parameters, we assigned the BED files of the ATAC-Seq peaks to promoter-transcriptional start site (TSS), transcription termination site (TTS), intron, intergenic and exon regions.

### ChIP library generation and sequencing

The following antibody was used for ChIP-seq experiments: anti-JUNB (3753). ChIP assays were conducted as previously described [29]. We prepared 2×10 ^7^ AGS-OR cells for nuclear extracts. The cells were lysed in RIPA buffer, and chromatin was sheared by sonication. At 4°C, the nuclear lysates were then incubated with protein A Dynabeads (Invitrogen, Carlsbad, USA) overnight with 3–5 μg of antibody for each sample. Following immunoprecipitation, the beads were recovered. At 55°C, the DNA was eluted, cross-links reverted, and then purified with a QIAquick PCR Purification Kit (Qiagen, Hilden, Germany). ChIP DNA was quantitated using the Qubit dsDNA HS assay and Qubit 3.0 Fluorometer (Thermo Fisher, Waltham, USA). Purified ChIP DNA (5‒10 ng) was used as input material for the sequencing library and sequenced using 150 bp paired-end reads on an Illumina HiSeq X platform (San Diego, USA).

### Identification of differentially accessible regions (DARs)

Compared to AGS-WT cells, if regions had average FC>1.5 in AGS-OR cells, we defined them as hyperaccessible regions. Conversely, if regions had average FC>1.5 in AGS-WT cells, we defined them as hypoaccessible regions.

### GO, KEGG and gene set enrichment analysis (GSEA) analysis

The enrichment of GO and KEGG was analyzed using the R package clusterProfiler [30]. To reveal the association between the top 20 pathways and DEGs in AGS-OR cells, cytoscape network analysis was used. The log2 ranked the genes (fold change) and GSEA v4.1.076 was performed using ranked lists with gene sets from MsigDB v7.4.

### TF motif enrichment

BEDTools (v.2.25.0) was used to obtain the peak summits overlapping with DARs, and findMotifsGenome was employed to find the known motif for discovering the binding for a center TF. We only selected motifs with *P* values of <0.01. HOMER (v4.10) was utilized to find the nearest genes surrounding the top 50 TFs and assess the probability of TFs with a 1 kb window around the peak summits [15].

### Spearman correlation of IC _50_ score and *JUNB* gene expression

The transcriptome data and corresponding clinical data of gastric cancer patients were from The Cancer Genome Atlas (TCGA) and Gene Expression Omnibus (GEO) database GSE51429. Chemotherapy responses were predicted through the public database “The Genomics of Drug Sensitivity in Cancer (GDSC)” ( https://www.cancerrxgene.org/). The prediction was performed using the R package “pRRophetic”. The IC _50_ values were estimated by ridge regression.

### Patients and tissue samples

A total of 281 patients who underwent D2 gastrectomy for GC at Zhongshan Hospital, Fudan University between January 2010 and June 2017 were recruited into the study. This study was approved by the Ethics Committee of Zhongshan Hospital affiliated with Fudan University. All patients were informed of the procedure and signed informed consent forms.

### Statistical analysis and data visualization

Data are presented as the mean±standard deviation. Both the generation of plots and statistical analyses were conducted with the R platform4 and GraphPad. Student’s *t* test and ANOVA were conducted for data comparison. Fold change, *P* values and false discovery rate (FDR) were calculated in the analysis, and relevant *P* values are indicated in each panel. The genomic signal changes were visualized with an IGV genome browser.

## Results

### Characterization of critical genes and pathway enrichment involved in acquired resistance to oxaliplatin

**Figure 1:**
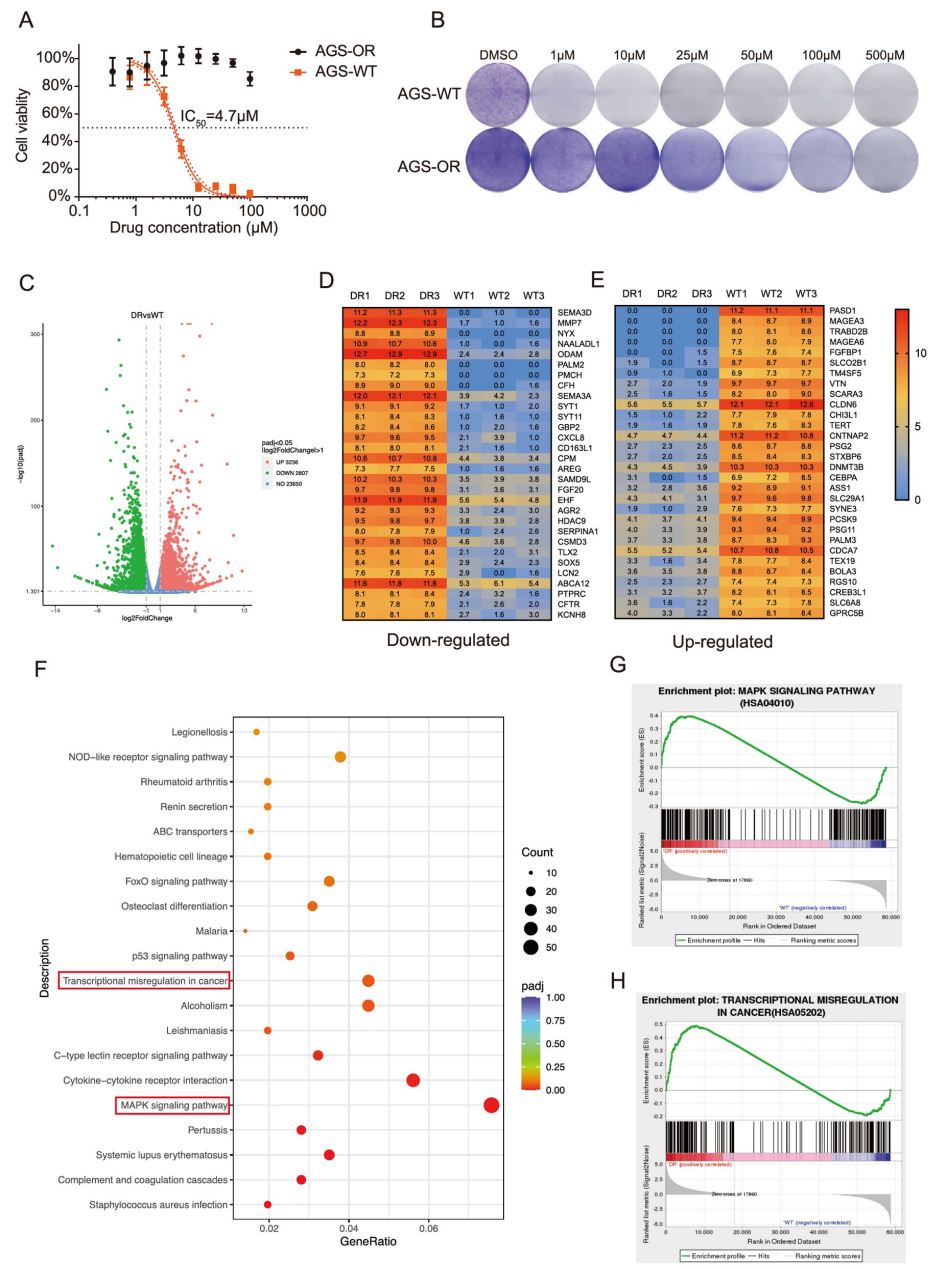
Figure 1 Identification of key genes and significant signaling pathways (A) The half-inhibitory concentration of AGS-WT and AGS-OR cells. (B) AGS-WT and AGS-OR cells were harvested for the colony-forming assay. Cells were exposed to different doses of oxaliplatin (0, 1, 10, 25, 50, 100 and 500 μM) for 10 days. (C) Differential analysis was conducted in AGS-WT and AGS-OR cells, and the differentially expressed genes (DEGs) are shown in a volcano plot. (D,E) The cluster of DEGs is shown in the heatmap. (F) The top 20 signaling pathways are shown in the bar chart. These pathways are significantly enriched in upregulated genes in AGS-OR cells. (G,H) GSEA suggested that oxaliplatin resistance in AGS-OR cells is positively related to the MAPK signaling pathway and transcriptional misregulation in cancer.

To understand the characteristics of oxaliplatin resistance, we first established AGS-OR cells *in vitro*. The IC _50_ value of oxaliplatin in AGS-WT cells was 4.7 μM, while for AGS-OR cells, the IC _50_ was beyond the range of concentrations tested ( Figure 1A), indicating a higher degree of oxaliplatin resistance in established resistant cells than in the parental cells. Furthermore, according to the colony formation assay, the number of colonies formed by AGS-WT cells was significantly decreased with increasing concentrations of oxaliplatin. The number of colonies formed by AGS-OR cells was unaffected by oxaliplatin treatment ( Figure 1B). Next, RNA sequencing was used to identify possible genes related to oxaliplatin resistance in AGS-OR cells. In total, 6043 differentially expressed genes (DEGs), including 3236 upregulated genes and 2807 downregulated genes, were identified ( Figure 1C,D). KEGG and GSEA analyses were then applied to find the crucial pathways, and the results showed that upregulated DEGs were primarily enriched in the MAPK signaling pathway, transcriptional misregulation in cancers, and the p53 signaling pathway ( Figure 1F–H).

### Chromatin accessibility changes at the genome-wide level

The role of epigenetic alterations, including changes in chromatin accessibility and in drug resistance, has been revealed in recent studies [ 31– 33] . Thus, we proposed that chromatin accessibility may contribute to oxaliplatin resistance. We performed an ATAC-seq experiment to assess the alteration of chromatin accessibility in AGS-WT and AGS-OR cells. As shown in Supplementary Figure S1A,B, a strong correlation between replicates and the coordinate distribution of fragment insert sizes of approximately 200 bp in the ATAC-seq data existed, which suggested that the ATAC-seq data were of good quality ( Supplementary Figure S1B). According to distribution analysis, distal intergenic regions had the highest percentage of accessible chromatin areas in the genome, followed by introns and promoters ( Supplementary Figure S1C).

**Figure 2:**
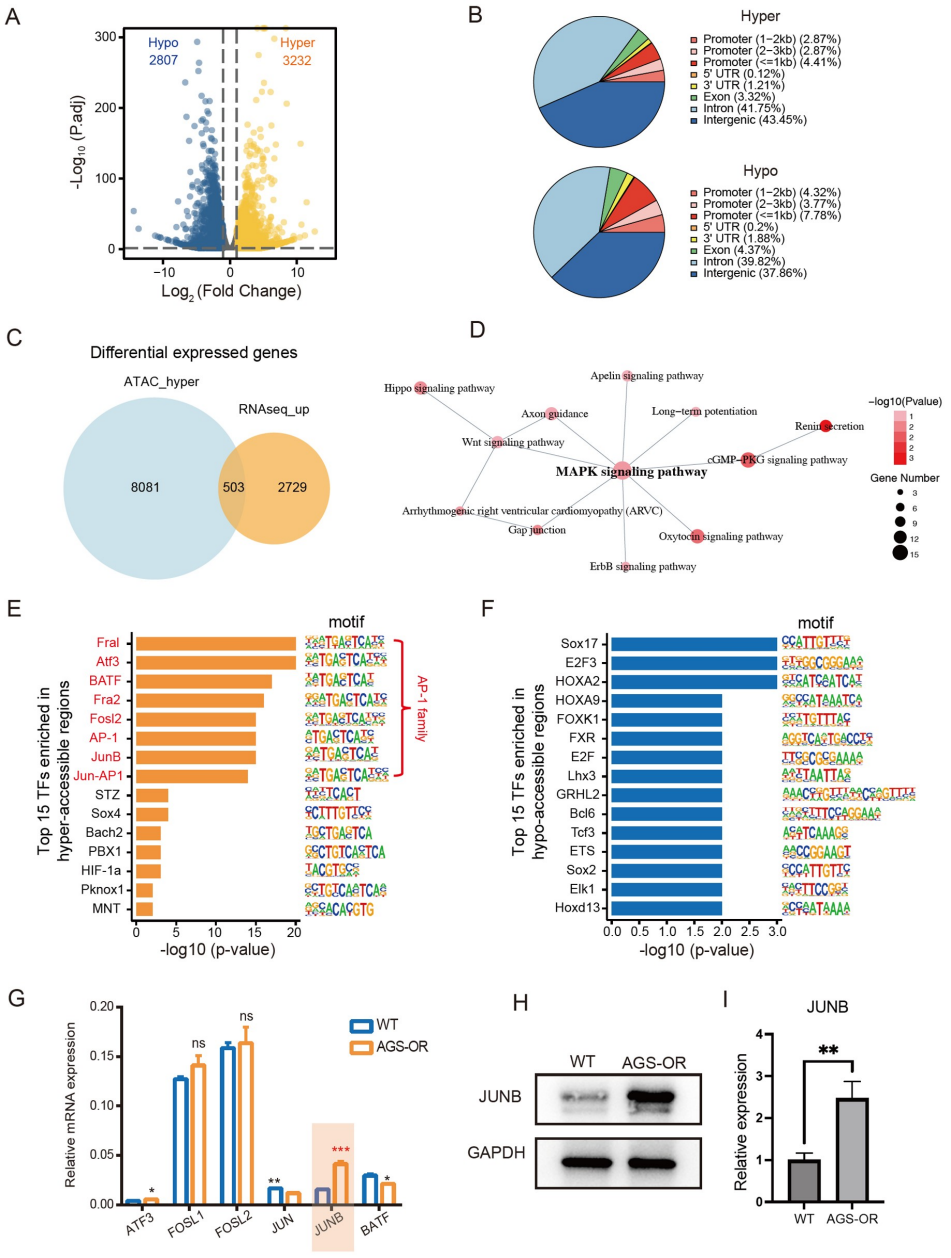
Figure 2 The association between the specific chromatin-accessible regions and gene expression in AGS-OR cells (A) Differential ATAC-seq peak analysis between AGS-WT and AGS-OR cells defined 3232 peaks that are greater in OR cells. (B) Pie chart illustrating the proportions of hyper and hypoaccessible sites in the following genomic regions: introns, exons, intergenic regions, 3′ and 5′ UTRs, and promoters. The promoter-TSS area is defined as peak peaks up to 1 kb upstream and 100 bp downstream of the TSS. (C) The overlap between DEGs and the nearby genes of hyperaccessible regions. (D) The network showing pathways significantly enriched by overlapping genes. (E,F) TF motif enrichment of DARs ( P values calculated from HOMER v4.10). (G) mRNA expression changes in AP-1 family genes. (H) High JUNB protein levels were detected in AGS-OR cells by western blot analysis. (I) Quantification of JUNB protein expression.

The accessibility discrepancies between the two cell lines were subsequently examined using differentially accessible peak analysis. Overall, 3232 areas became more accessible in AGS-OR cells than those in AGS-WT cells. In contrast, 2807 areas were less accessible in the AGS-OR cells; we labelled these chromatin regions as hyperaccessible and hypoaccessible ( Figure 2A). These regions are primarily intragenic and distal to the genes that they control ( Figure 2B), with only a small number of promoter-proximal regions displaying differential accessibility. This finding raised the possibility that DARs are distal regulatory elements such as enhancers. We annotated differentially accessible sites to the closest genes in accordance with positions in the Hg38 genome to assess the possible connections between DARs and DEGs. By overlapping upregulated DEGs and genes nearest to the hyperaccessible loci, we identified 503 intersecting genes ( Figure 2C). Consistent with the transcriptome analysis, these genes were then subject to KEGG pathway analysis and the results showed that the MAPK signaling pathway was substantially enriched ( Figure 2D).

### Identification of oxaliplatin resistance-associated TFs

Previous studies revealed that TFs may bind to motifs in promoter regions and enhancer regions of target genes, requiring an open chromatin conformation, to regulate their expression during cancer development [ 34, 35] . To examine TF motifs that could be present in AGS-OR cells, we examined chromatin regions that had higher accessibility and were related to genes with higher mRNA expression in AGS-OR cells than in parental cells. We used the motif discovery software HOMER to assess the potential TFs located in the DARs of AGS-OR cells. By integrating known TF motifs and chromatin openness information in ATAC-seq data, we predicted TF occupancy on chromatin across the entire genome. In DARs, to identify the TF motif occurrences and binding sites, we searched the flanking sequences of 200 bp surrounding ATAC-seq peak summits.

In AGS-OR cells, the top 15 TF candidates were listed in terms of enrichment in hyperaccessible ( Figure 2E) or hypoaccessible regions ( Figure 2F). Moreover, in hyperaccessible regions, 8 of the 15 significantly enriched TFs were from the AP1 family; these included Fral, Atf3, BATF, Fra2, Fosl2, AP-1, JUNB, and JUN-AP1. Therefore, we think that the AP1 family may play an essential role in regulating oxaliplatin resistance in GC. To further clarify the specific regulator of the AP1 family in AGS-OR cells, we examined the mRNA levels of these TFs in AGS-WT and AGS-OR cells ( Figure 2G). Only *JUNB* was significantly upregulated in AGS-OR cells, and the results were further validated at the protein level by western blot analysis ( Figure 2H,I).

### Silencing of *JUNB* restores oxaliplatin sensitivity and high JUNB levels are correlated with a poor prognosis in GC patients

**Figure 3:**
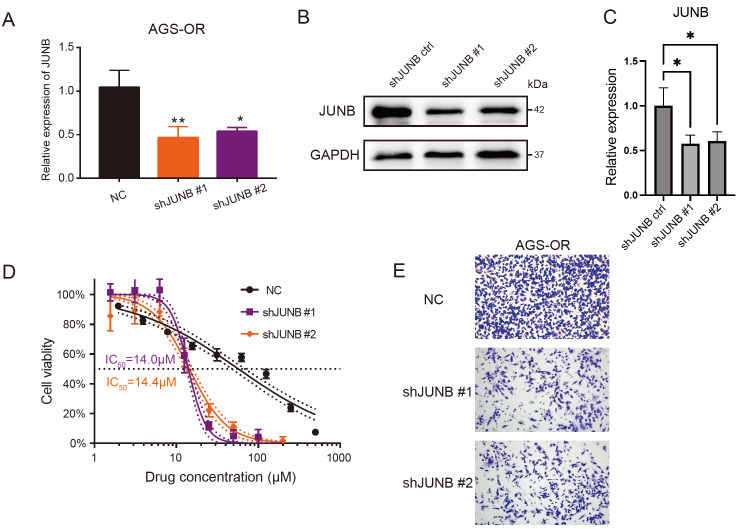
Figure 3 Silencing of *JUNB* restores oxaliplatin sensitivity and suppresses cell metastasis (A,B) Verification of JUNB silencing in AGS-OR cells at the mRNA and protein levels. Data are presented as the mean±standard deviation. (C) Quantification of JUNB protein expression. (D) The IC 50 values of JUNB-NC and JUNB-silenced AGS-OR cells. NC, negative control. (E) Representative images of the transwell assay. A total of 50,000 cells were seeded per well in 24-well plates and cultured for 24 h.

To determine the function of JUNB in oxaliplatin-resistant GC cells, we stably silenced *JUNB* by lentiviral shRNA transfection ( Figure 3A–C). The IC _50_ values of oxaliplatin in the control and *JUNB*-knockdown AGS-OR cells were measured, and the IC _50_ values were significantly reduced when *JUNB* was silenced ( Figure 3D), which implied that a high level of JUNB confers oxaliplatin resistance in GC. Moreover, transwell assays were applied to examine cell metastasis, and silencing of *JUNB* significantly impaired the metastasis of AGS-OR cells ( Figure 3E).

**Figure 4:**
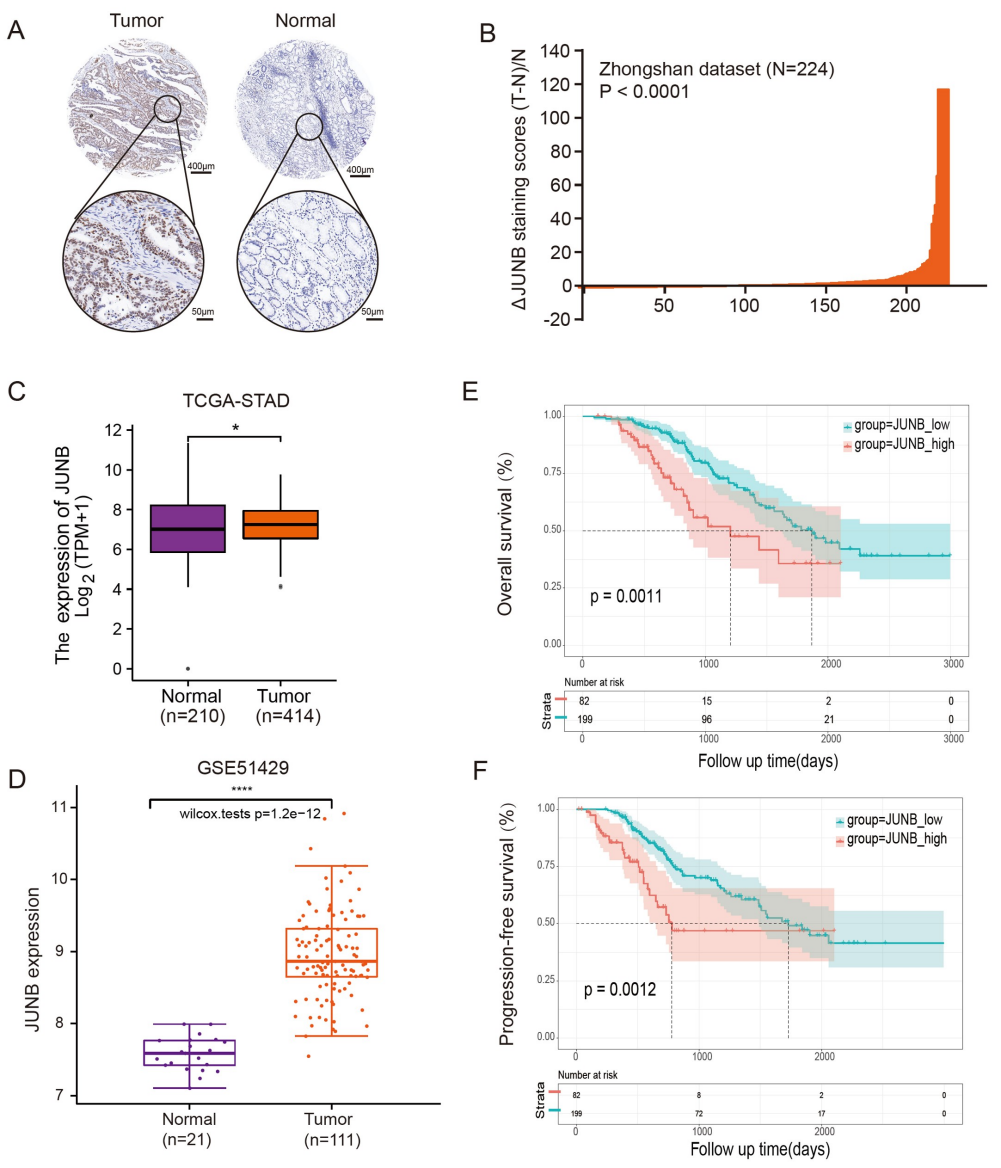
Figure 4 High JUNB levels are correlated with a poor prognosis in GC patients (A) Representative image of immunohistochemical staining for JUNB. (B) The distribution of the difference in JUNB immunoreactivity score [ΔScores=(tumor–normal)/normal]. The ΔScores of JUNB staining were available in 224 pairs of tissues. (C,D) JUNB expression was upregulated in colon cancer based on samples from TCGA-STAD and GEO database GSE51429. (E,F) Kaplan-Meier curves suggested that patients with upregulated JUNB had a shorter overall survival (OS) time and progression-free survival (PFS) time in the Zhongshan cohort.

To further evaluate the clinical significance of JUNB, we analyzed several cohorts and found that JUNB expression was much higher in tumor tissues than in normal tissues in both the TCGA-GC dataset and the GSE51429 dataset ( Figure 4C,D). In addition, the elevated expression of JUNB was confirmed again in a microarray assay of GC samples ( *n*=224; *P*<0.001; Figure 4A,B). Kaplan-Meier analysis further demonstrated that high expression of JUNB was correlated with worse overall survival (OS) and progression-free survival (PFS) for gastric cancer patients from the Zhongshan-GC dataset ( Figure 4E,F).

### Validation of *JUNB* regulation of MAPK signaling pathway-related genes

**Figure 5:**
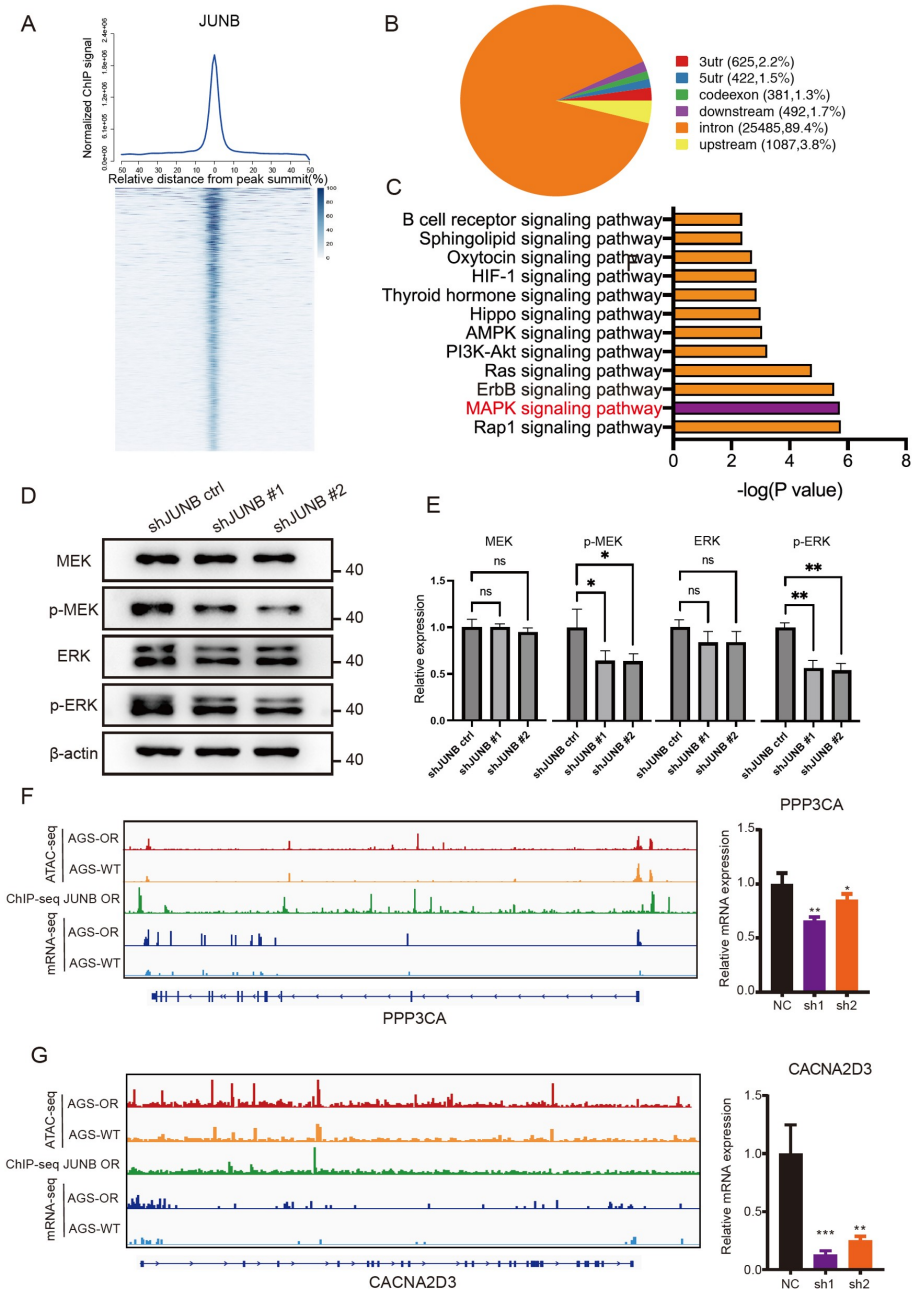
Figure 5 Validation of *JUNB* regulation of MAPK signaling pathway-related genes (A) ChIP-Seq summary plot of JUNB-binding intensities across JUNB peaks in AGS-OR cells. (B) The distributions of JUNB-binding regions are shown in the pie charts. (C) Pathways significantly enriched by specific downstream targets controlled by JUNB. (D) Western blot analysis of MEK1/2, pMEK1/2, Erk1/2, and p-Erk1/2 in JUNB-knockdown cells. The β-actin protein was used as an internal control. (E) Quantification of relative protein expression. (F,G) IGV browser demonstrated signals of ATAC-seq, ChIP-seq and mRNA-seq of PPP3CA and CACNA2D3. RT-qPCR assay confirmed the downregulation of PPP3CA and CACNA2D3 expressions in the JUNB-knockdown group.

ChIP-seq was used to identify specific downstream targets controlled by JUNB and 28,492 peaks were discovered in comparison to the input signals ( Figure 5A). The pie charts display the JUNB-binding distributions. JUNB predominantly binds to distal intergenic areas, which is consistent with the aforementioned ATAC findings ( Figure 5B). The MAPK and Rap1 signaling pathways were shown to be the most enriched crosstalk functions, followed by the ERBB signaling pathway and Ras signaling pathway, according to the KEGG analysis ( Figure 5C). Taken together with previous RNA-seq data, these results indicated that JUNB may induce oxaliplatin resistance via the MAPK signaling pathway. The protein levels of p-MEK1/2 and p-Erk1/2 were decreased after *JUNB* knockdown, indicating the inhibition of MAPK signaling ( Figure 5D,E). As shown in Figure 5F,G and Supplementary Figure S2A–D, in the AGS-OR cells, JUNB was present near the loci of *PPP3CA*, *MET*, *CACNA2D3*, *EREG*, *FGF20*, and *PTPR*R, which are key genes involved in MAPK signaling regulation, followed by changes to the accessible regions and higher mRNA expression compared with parent cells. Therefore, these results suggest that JUNB regulates the activity of the MAPK pathway by binding to the TSS of key genes of the MAPK pathway and causing changes in their chromatin accessibility to maintain oxaliplatin resistance in AGS-OR cells.

### Combination with MAPK inhibitors is a potential strategy to restore oxaliplatin sensitivity in oxaliplatin-resistant GC cells

**Figure 6:**
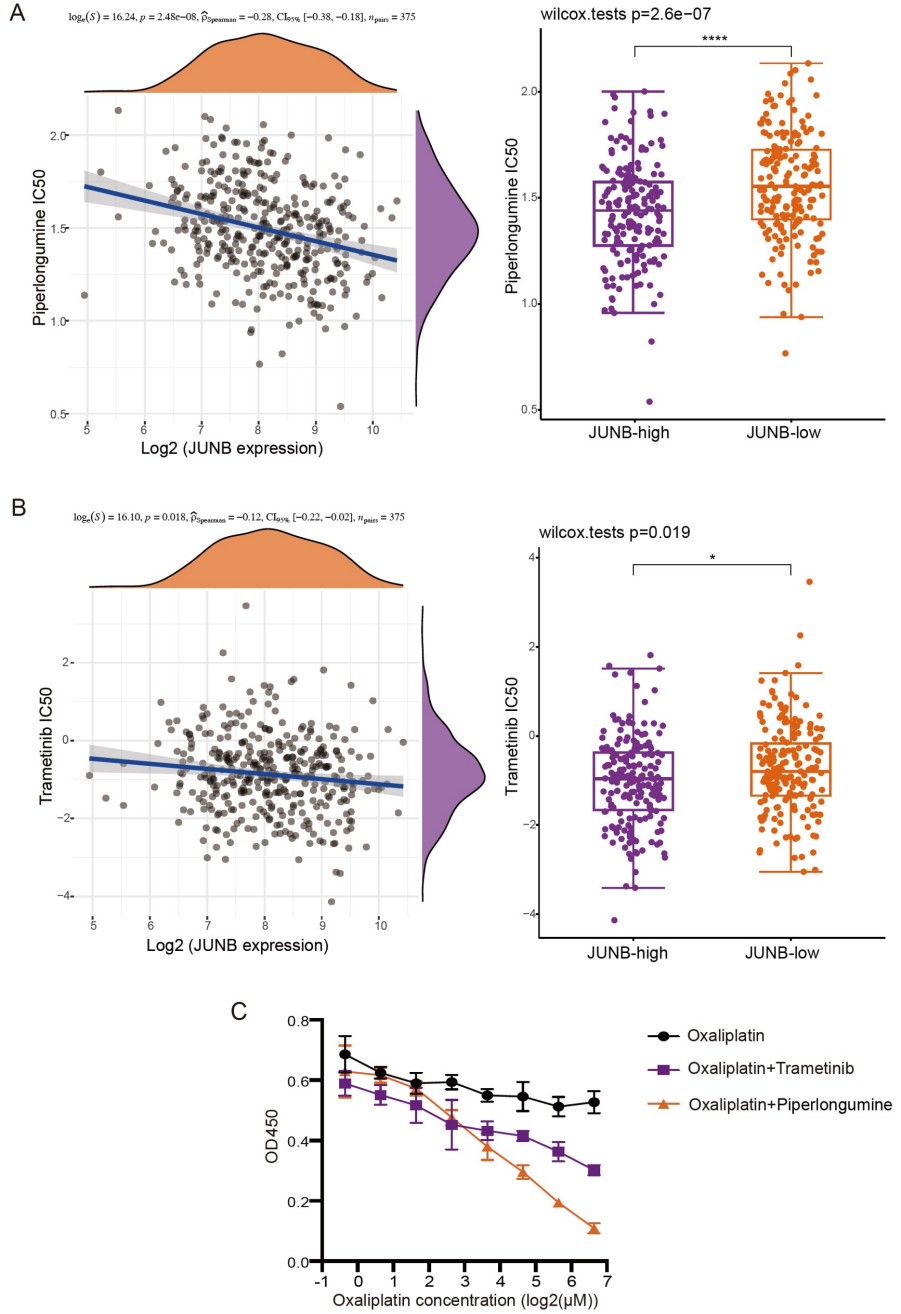
Figure 6 Combination with MAPK inhibitors is a potential strategy to restore oxaliplatin sensitivity in oxaliplatin-resistant GC cells (A,B) The negative correlation between the expression of JUNB and the IC 50 score of piperlongumine and trametinib was confirmed by Spearman correlation analysis. The ordinate shows the distribution of the IC 50 score, and the abscissa shows the various expression levels of the samples (piperlongumine: P=2.48×10 –8, ρ=–0.28, n=375; trametinib: P=0.018, ρ=–0.12, n=375). (C) CCK8 analysis of AGS-OR cells treated with a gradient concentration of oxaliplatin and oxaliplatin combined with low-dose piperlongumine and trametinib.

Finally, we were able to develop a combination treatment to combat oxaliplatin resistance in GC. First, the response of TCGA samples to the MAPK pathway inhibitors piperlongumine and trametinib was predicted using GDSC public data. Intriguingly, samples with higher JUNB expression showed lower piperlongumine or trametinib IC _50_ values, indicating that they may be more susceptible to the antitumour actions of these medications ( Figure 6A,B). The IC _50_ values of piperlongumine and trametinib in AGS-OR cells were 4.31 μM and 0.05 μM, respectively ( Supplementary Figure S3A,B). To test whether they can reverse oxaliplatin resistance, we used low concentrations of the ERK inhibitor piperlongumine or MEK inhibitor trametinib in combination with oxaliplatin. The standard for concentration selection is an effective inhibition rate of approximately 20%. The combination of oxaliplatin and these two MAPK inhibitors in AGS-OR cells demonstrated cooperative effects ( Figure 6C), indicating that the combined strategy could effectively reverse oxaliplatin resistance in GC cells.

## Discussion

Oxaliplatin is widely used as the first-line chemotherapeutic drug for GC. However, due to the lack of effective predictive markers of sensitivity to treatment, the response is not satisfactory, and oxaliplatin resistance is still currently one of the major obstacles in GC chemotherapy [36]. Thus, it is of great significance to clarify the molecular mechanism of oxaliplatin resistance in reversing drug resistance in cancer therapy. To further investigate the mechanism of GC resistance, the AGS-OR cell line was first established in this study, and with applicable multiomics data from ATAC-Seq, RNA-Seq, and ChIP-seq, the integrative analysis approach used here demonstrated that the core TF JUNB is the key regulator in the gene regulatory network of AGS-OR cells.

JUNB, also known as AP-1 transcription factor subunit, is a protein-coding gene that is located in the nucleoplasm. It enables sequence-specific dsDNA binding activity and exerts distinct functions depending upon the cellular origin, cell-cycle stage, and environmental conditions [11]. Previous studies have indicated that JUNB is a critical determinant of myelopoiesis [ 37, 38] and several lymphomas [ 39– 41] . Studies of solid tumors also demonstrated JUNB as a potential target in renal cell carcinoma [42], cervical cancer [43], endometrial cancer [44] and colorectal cancer [45], while its role in GC is not yet clear.

In our study, Kaplan-Meier curves suggested that JUNB had the potential to identify patients with poorer OS and PFS in GC. We further confirmed that JUNB could repress the proliferation and promote the migration of GC cells and enhance the tolerance of AGS cells to different concentrations of oxaliplatin. Oxaliplatin is a platinum-based antineoplastic agent that functions by forming inter- and intrastrand DNA-platinum adducts specifically in cancer cells, inhibiting gene transcription, which leads to cell death [46]. Therefore, tumors fight back by enhancing DNA damage repair, resulting in oxaliplatin resistance [47]. As a transcription-induced negative regulator, JUNB is recruited into existing transcriptional complexes of immediate early genes to attenuate their transcriptional activity; thus, we suggested that inhibiting the expression of JUNB could prevent the activation of DNA damage repair.

JUNB induces oxaliplatin resistance and promoted invasion and metastasis by binding to the TSS of regulated genes of the MAPK signaling pathway, including *PPP3CA*, *CACNA2D3*, *EREG*, and *FEF20*, thus activating the MAPK signaling pathway. Combined treatment with ERK and MEK inhibitors partly reverses oxaliplatin sensitivity in oxaliplatin-resistant cells.

Our study serves as a starting point for further understanding different pathways in tumor drug resistance and mechanistic studies to validate their roles. While additional studies will need to be conducted, as our study was performed in a single cell line model and may not represent the overall conclusions, our results still provide a strong foundation for such translational research. Another problem worth pointing out is that AGS is a p53 wild-type cell line, which can only represent less than half of gastric cancers. In the future, it is necessary to establish oxaliplatin-resistant strains in p53 mutant cells to improve the study of related drug resistance mechanisms.

Overall, our results elucidate the potential mechanisms underlying JUNB upregulation in GC cells and its contribution to oxaliplatin resistance. Oxaliplatin resistance following JUNB upregulation is critically dependent on binding to the TSS of MAPK signaling pathway-regulated genes and can be reversed by MAPK pathway inhibitors. The findings in this study highlight the potential of therapeutics targeting JUNB in oxaliplatin resistance and support further clinical development of combination therapy with oxaliplatin and MAPK inhibitors for the treatment of advanced oxaliplatin-resistant patients with GC.

## Supporting information

23065Graphical_abstract

## Supplementary Data

Supplementary data is available at *Acta Biochimica et Biophysica Sinica* online.
