# Supplementary figures and images for: JUNB mediates oxaliplatin resistance via the MAPK signaling pathway in gastric cancer by chromatin accessibility and transcriptomic analysis: JUNB mediates oxaliplatin resistance in gastric cancer
Source: Acta Biochim Biophys Sin (Shanghai). 2023 Jun 19;55(11):1784–96. doi: 10.3724/abbs.2023119 (PMC10679881; doi:10.3724/abbs.2023119)

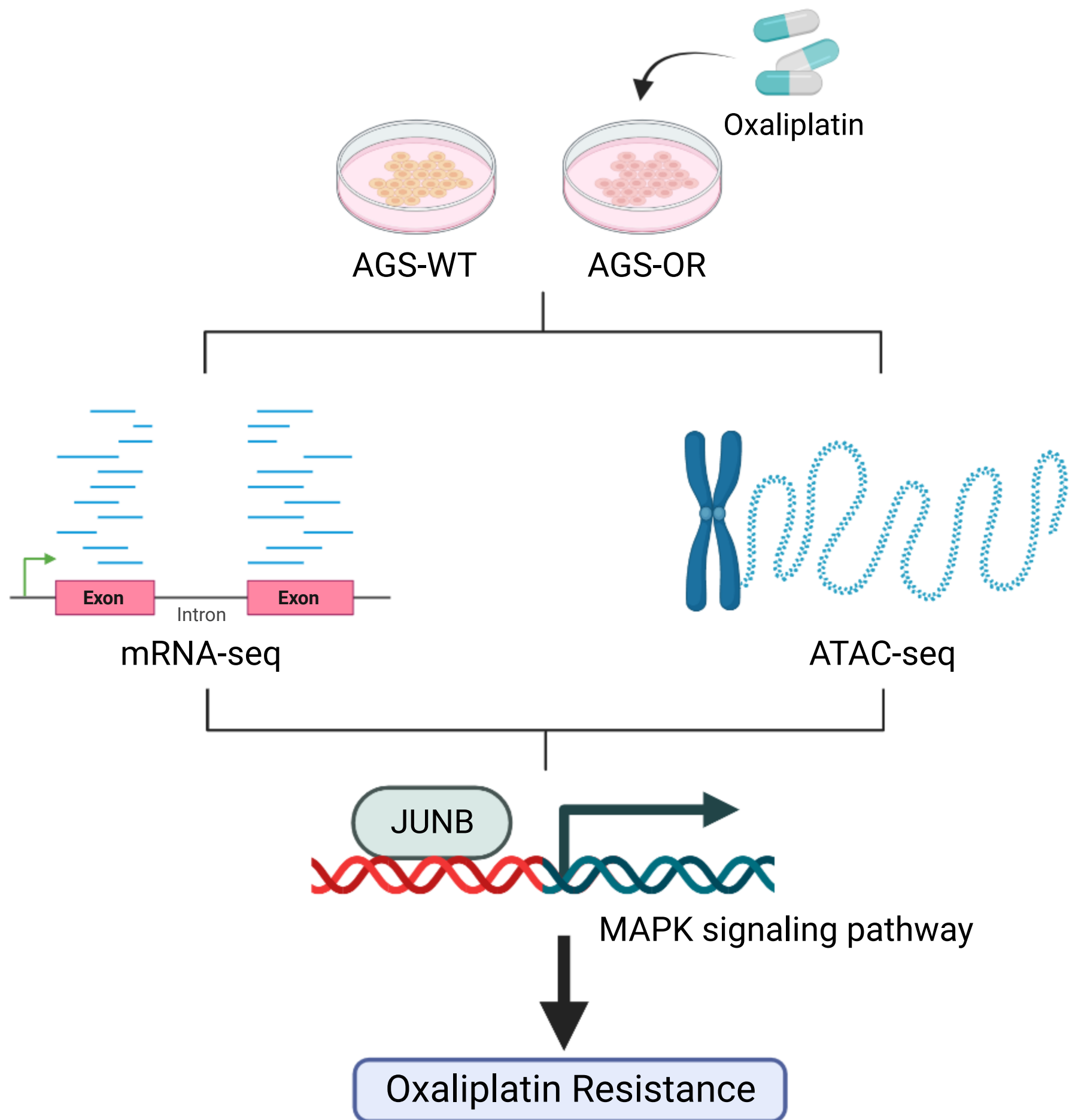

Supplement: 23065Graphical_abstract [file 23065Graphical_abstract.pdf]
